# Serotonin Mediates Depression of Aggression After Acute and Chronic Social Defeat Stress in a Model Insect

**DOI:** 10.3389/fnbeh.2018.00233

**Published:** 2018-10-08

**Authors:** Jan Rillich, Paul A. Stevenson

**Affiliations:** Institute for Biology, Leipzig University, Leipzig, Germany

**Keywords:** aggression, agonistic behavior, subjugation, loser effect, 5HT, nitric oxide, dopamine, octopamine

## Abstract

In all animals, losers of a conflict against a conspecific exhibit reduced aggressiveness, often coupled with depression-like symptoms, particularly after multiple defeats. While serotonin (5HT) is involved, discovering its natural role in aggression and depression has proven elusive. We show how 5HT influences aggression in male crickets, before, and after single and multiple defeats using serotonergic drugs, at dosages that had no obvious deleterious effect on general motility: the 5HT synthesis inhibitor alpha-methyltryptophan (AMTP), the 5HT_2_ receptor blocker ketanserin, methiothepin which blocks 5HT receptor subtypes other than 5HT_2_, 5HT's precursor 5-hydroxytryptophan (5HTP) and re-uptake inhibitor fluoxetine. Contrasting reports for other invertebrates, none of the drugs influenced aggression at the first encounter. However, the recovery of aggression after single defeat, which normally requires 3 h in crickets, was severely affected. Losers that received ketanserin or AMTP regained their aggressiveness sooner, whereas those that received fluoxetine, 5HTP, or methiothepin failed to recover within 3 h. Furthermore, compared to controls, which show long term aggressive depression 24 h after 6 defeats at 1 h intervals, crickets that received AMTP or ketanserin regained their full aggressiveness and were thus more resilient to chronic defeat stress. In contrast, 5HTP and fluoxetine treated crickets showed long term aggressive depression 24 h after only 2 defeats, and were thus more susceptible to defeat stress. We conclude that 5HT acts after social defeat *via* a 5HT_2_ like receptor to maintain depressed aggressiveness after defeat, and to promote the susceptibility to and establishment of long-term depression after chronic social defeat. It is known that the decision to flee and establishment of loser depression in crickets is controlled by nitric oxide (NO), whereas dopamine (DA), but not octopamine (OA) is necessary for recovery after defeat. Here we show that blocking NO synthesis, just like ketanserin, affords resilience to multiple defeat stress, whereas blocking DA receptors, but not OA receptors, increases susceptibility, just like fluoxetine. We discuss the possible interplay between 5HT, NO, DA, and OA in controlling aggression after defeat, as well as similarities and differences to findings in mammals and other invertebrate model systems.

## Introduction

Aggression toward a conspecific is a widespread behavioral strategy in the Animal Kingdom adapted to secure resources and ensure survival at minimal cost (Stevenson, 2018). In addition to the physical dangers, losing a conflict (social defeat) can have enduring adverse behavioral costs, including suppressed aggressiveness (De Boer et al., 2016) often coupled with general depression like symptoms, particularly after chronic social defeat (rodents Hammels et al., 2015; Koolhaas et al., 2017; fish: Backstrom and Winberg, 2017; insects: Rose et al., 2017; Trannoy and Kravitz, 2017; crayfish: Bacque-Cazenave et al., 2017). Social defeat is thus currently viewed as a model for gaining insights into depression in humans (Laman-Maharg and Trainor, 2017), improved animal welfare (Toyoda, 2017) and behavioral syndromes underlying animal “personality” (Briffa et al., 2015).

The proximate mechanisms underlying defeat associated depression are not fully understood. Aggressive experience modifies numerous neurotransmitter systems (De Boer et al., 2016), and drugs that influence them have manifold effects on aggression (Trainor et al., 2017). Among them, serotonin (5HT) has a complex relationship to aggression that depends on age, sex and social status, which reflects the intricacy of the 5HT system with its many receptor subtypes and widespread innervation (Carhart-Harris and Nutt, 2017). Generally, however, 5HT precursors, re-uptake inhibitors and 5HT receptor agonists typically reduce overt aggression in vertebrates including man (De Boer et al., 2016; Carhart-Harris and Nutt, 2017; Trainor et al., 2017). Serotonin is thus thought to dampen aggression by promoting withdrawal or terminating aggression (Olivier, 2015). However, 5HT drugs can also increase aggression, by decreasing submissiveness after social defeat (Morrison and Cooper, 2012; Bauer, 2015; Clinard et al., 2015; Olivier, 2015). Thus, 5HT is a potential mediator of loser depression, chronic defeat stress and general stress (Hammels et al., 2015; Koolhaas et al., 2017). However, the extent to which this occurs normally, or only under extreme, pathological conditions, is questioned (Olivier, 2015).

Notwithstanding remarkable similarities in the mechanisms underlying aggression in insects, mice and man (Thomas et al., 2015), the role proposed for 5HT seems to differ. In invertebrates, 5HT, its precursor, 5HT_1A_ agonists and genetic activation of 5HT neurons can increase aggression and win chances while reducing the tendency to flee in crustaceans (Kravitz, 2000), fruit flies (Dierick and Greenspan, 2007; Johnson et al., 2009; Alekseyenko et al., 2010) and stalk eyed flies (Bubak et al., 2014). Recently though, blockade of 5HT receptors was reported to prohibit the acquisition of anxiety like behavior after defeat in crayfish (Bacque-Cazenave et al., 2017). Furthermore, 5HT neurons in insects (*Drosophila*) also have inhibitory effects on behavior (Pooryasin and Fiala, 2015) and mediate stress induces behavioral depression (Ries et al., 2017), but it is not known if 5HT influences post defeat depression.

Here we investigate how 5HT drugs affect aggression in crickets after single and multiple defeats and compare this with their action before losing in socially naive crickets. At present, the role of 5HT in the aggressive behavior of crickets is unclear (reviewed in Stevenson and Rillich, 2017). Inhibition of 5HT synthesis is claimed to reduce win chances (Dyakonova et al., 1999), but have no clear effect on aggressiveness *per se* (Stevenson et al., 2000, 2005), whereas 5HT's precursor enhances some elements of cricket aggression (e.g., fight duration), but reduces others (e.g., attack frequency), without altering win chances (Dyakonova and Krushinsky, 2013). It has thus been suggested that “behavioral features of dominant male crickets are likely to be connected with the activation of the serotonergic system” whereas “a decrease in serotonergic activity may be functionally important for the control of loser behavior” (Dyakonova and Krushinsky, 2013). More recently, it was found in crickets that nitric oxide (NO) triggers the actual decision to flee and establishes subsequent loser depression (Stevenson and Rillich, 2015; Rillich and Stevenson, 2017), whereas octopamine and dopamine (OA, DA, Stevenson et al., 2005; Rillich and Stevenson, 2014) promote recovery. In view of this, we also test how drugs that influence these neuromodulators influence aggression after chronic social defeat in comparison to serotonergic drugs. Our experiments provided evidence that 5HT acts primarily in crickets to maintain depressed aggressiveness in losers after defeat, and particularly so after multiple defeat, most likely as the result of interactions with NO and DA. Our work thus provokes new thought on the roles of 5HT and NO in controlling aggression in insects and mammals.

## Materials and methods

### Experimental animals

Mature, 2–3 week-old, adult male crickets, *Gryllus bimaculatus* were taken from a breeding stock kept under standard conditions at Leipzig University (22–24°C, relative humidity 40–60%, 12 h: 12 h light: dark regime daily feeding on bran and vegetables). Prior to experimentation, they were isolated in glass jars with ample food and water for 48 h. All experiments were performed during daytime. All treatments complied with the Principles of Laboratory Animal Care and the German Law on the Protection of Animals.

### Evaluation of aggression

The aggressiveness of test crickets was evaluated by matching them against equally sized males (<5% weight difference) that were made hyper-aggressive by flying them in a wind stream before the match (as in Stevenson and Rillich, 2015) and which always won the contests. Contests were staged in a Perspex-glass arena (16 × 9 × 7 cm) and follow a stereotyped sequence which we score 0–6 to denote the level of aggressive escalation (Stevenson et al., 2000): Level 0: mutual avoidance. Level 1: one cricket attacks, the other retreats. Level 2: antennal fencing. Level 3: mandible threat by one cricket. Level 4: mandible threat by both. Level 5: mandible engagement. Level 6: grappling, an all-out fight. Fight duration, from initial contact to retreat of the loser, was recorded with a stopwatch, deducting any pauses that occasionally occurred.

### Pharmacological treatments

We tested the following drugs (Sigma Aldrich, Deisenhofen, Germany), which were injected into the haemocoel *via* the pronotal shield using a microsyringe: The 5HT-receptor antagonists ketanserin (+)-tartrate and methiothepin mesylate, which have differing receptor-subtype affinities (Vleugels et al., 2015); the competitive serotonin synthesis inhibitor alpha-methyltryptophan (AMTP); serotonin's precursor 5-hydroxytryptophan (5HTP) and re-uptake inhibitor fluoxetine hydrochloride; the selective octopamine-receptor blocker epinastine hydrochloride (Roeder et al., 1998); the insect dopamine-receptor blocker fluphenazine dihydrochloride (Degen et al., 2000); the inhibitor of nitric oxide (NO) production *N*_ω_-nitro-L-arginine methyl ester hydrochloride (LNAME) and its inactive enantiomer DNAME as control. Drugs were dissolved in either insect saline (contents in mM: NaCl 140, KCl 10, CaCl2 7, NaHCO3 8, MgCl2 1, N-trismethyl-2-aminoethanesulfonic acid 5, d-trehalose dihydrate, pH 7.4) or first in dimethylsulfoxide (DMSO) and diluted in ringer. The drug dosages used here are given in Table 1. That used for AMTP has been shown to be the minimum required to achieve almost complete depletion of serotonin as determined by immunocytochemistry, but above that shown to achieve complete depletion as determined by HPLC (see Stevenson et al., 2000). The dosages for all other drugs were selected as the minimum that induced clear effects on aggression, without obvious detrimental effect on general motility as judged by eye and were established in previous investigations (Rillich and Stevenson, 2014, 2015, 2017; Stevenson and Rillich, 2015), or in pilot experiments for the present study (Figure S1).

**Table 1:** Drugs, actions, and effective dosages without detrimental effects on motility.

**Drug**	**Action**	**Vehicle**	**μl, mM**	**μg**	**μg/g wt**.
ketanserin	5HT-receptor blocker	5% DMSO	20, 10	109	81
methiothepin	5HT-receptor blocker	5% DMSO	20, 10	91	67
AMTP	5HT synthesis inhibitor	Ringer	40, 100 × 3	873 × 3	646 × 3
5HTP	5HT precursor	Ringer	20, 5	22	16
fluoxetine	5HT re-uptake inhibitor	5% DMSO	20, 1	7	5
fluphenazine	DA-receptor blocker	5% DMSO	20, 10	102	76
epinastine	OA-receptor blocker	5% DMSO	20, 10	57	42
LNAME	NO synthesis inhibitor	Ringer	20, 10	54	40
DNAME	inactive form of LNAME	Ringer	20, 10	54	40

*They were injected 60 min prior to testing, except AMTP which was given on day 3, 2, and 1 prior to testing (as in Stevenson et al., 2000). Controls received vehicle or DNAME*.

### Procedure

To avoid possible temporal variations, we performed separate controls for each drug in parallel at approximately the same times. We ran 3 different protocols.

**Figure 1:**
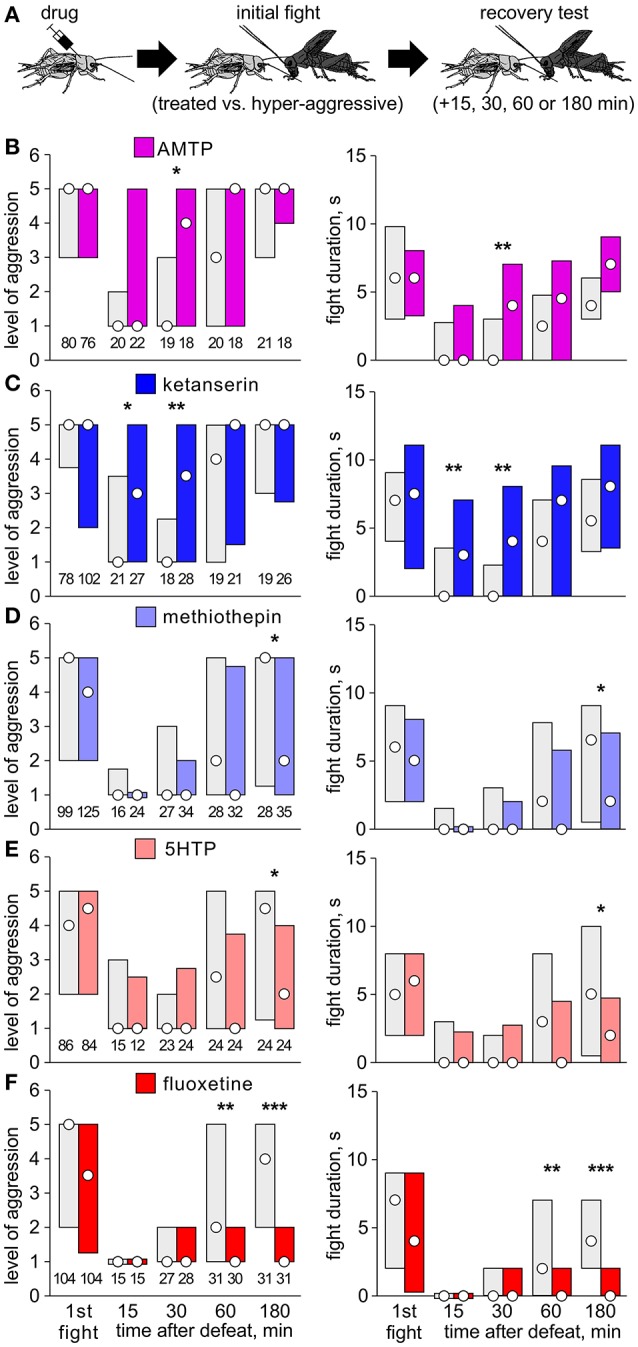
Effect of serotonergic drugs on initial fights and loser depression. **(A)** Procedure: Test crickets received vehicle or drug 60 min before an initial fight against a hyper-aggressive opponent. Separate cohorts of losers were then rematched at different times after defeat. **(B–F)** Level of aggression (left) and fight duration (right, circle: median, bars: interquartile range, IQR; *n* is given above the left x-axis) for crickets that received vehicle (gray bars) or **(B)** AMTP (violet bars); **(C)** ketanserin (dark blue bars); **(D)** methiothepin (light blue bars); **(E)** 5HTP (pink bars); **(F)** fluoxetine (red bars). Asterisks indicate significant differences between test and control groups as given by Mann-Whitney U-tests (*, **, ***: *p* < 0.05, 0.01, 0.001 respectively, otherwise not significant).

**Figure 2:**
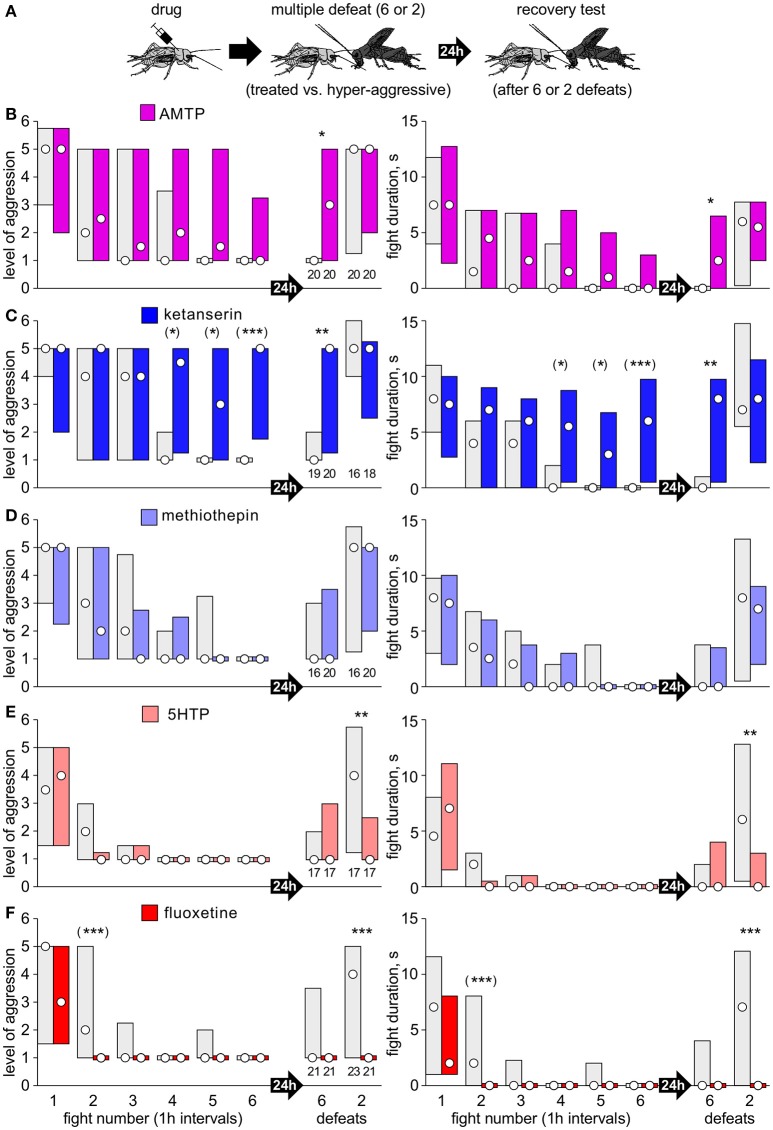
Serotonergic drugs and chronic social defeat. **(A)** As in Figure 1, except that separate cohorts of losers were rematched either 6 or 2 times in succession at 1 h intervals and once more 24 h later to check for long-term aggressive depression. **(B–F)** Bar charts giving the level of aggression (left) and fight duration (right, circle: median, bars: interquartile range, IQR; *n* is given above the left x-axis) for crickets that received: **(B)** AMTP (violet bars); **(C)** ketanserin (dark blue bars); **(D)** methiothepin (light blue bars); **(E)** 5HTP, (pink bars); **(F)** fluoxetine (red bars). Controls received vehicle (gray bars). Asterisks indicate significant differences between test and control groups as given by Mann-Whitney U-tests for the tests after 24 h after defeat: *, **, ***: *p* < 0.05, 0.01, 0.001. For the six repeated defeats, we applied the Bonferroni corrections for multiple comparisons: (*), (**), (***): *p* < 0.01, 0.002, 0.0002).

**Figure 3:**
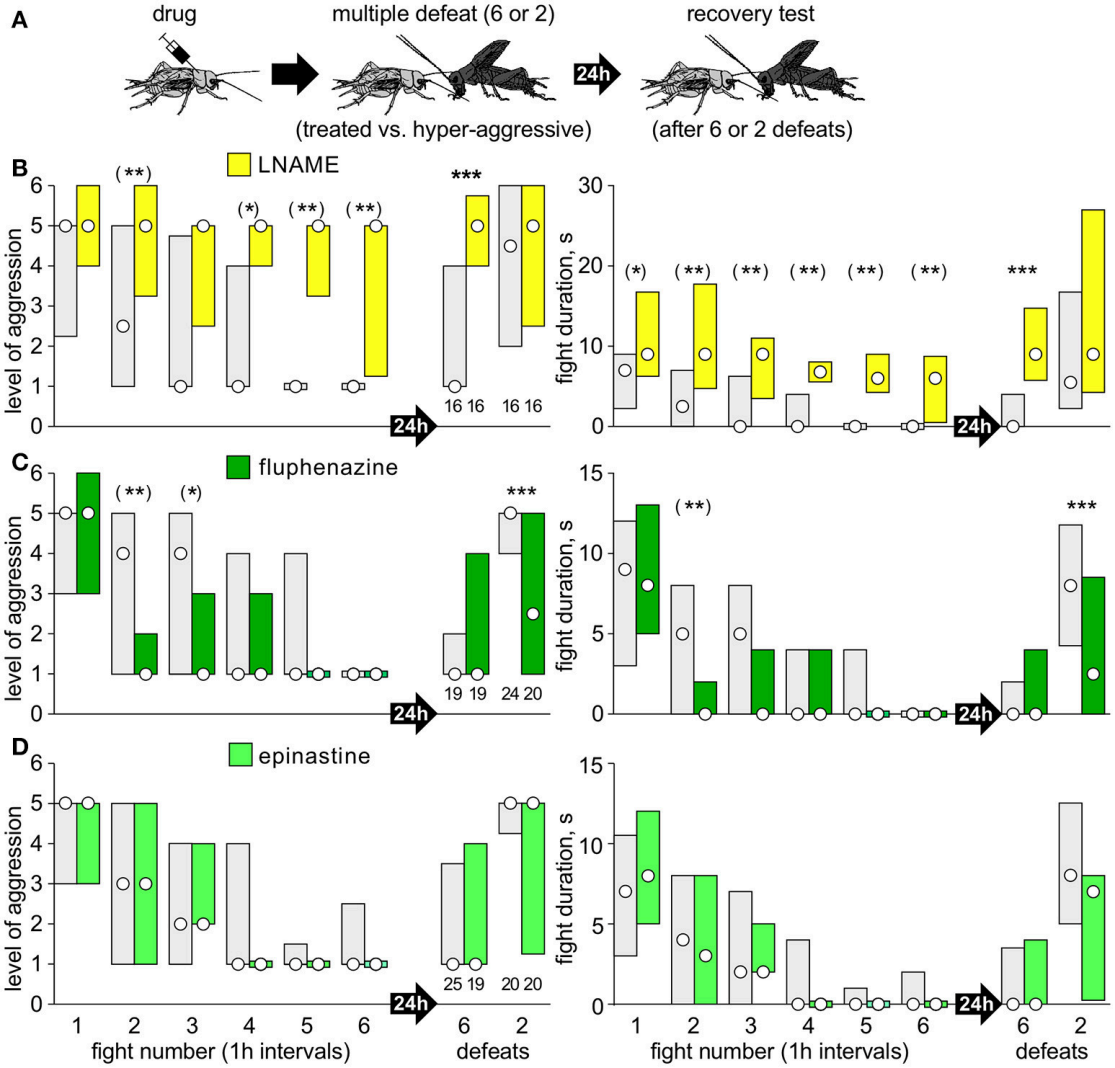
Test for effects of NO, DA, and OA. **(A)** Procedure, as for Figure 2, but with different drugs: **(B)** LNAME (yellow bars); **(C)** fluphen azine (dark green bars); **(D)** epinastine (light green bars). Controls received vehicle or DNAME to control for LNAME (gray bars). Asterisks indicate significant differences between test and control groups as given by Mann-Whitney U-tests for the tests after 24 h after defeat: *, **, ***: *p* < 0.05, 0.01, 0.001. For the six repeated defeats, we applied the Bonferroni corrections for multiple comparisons: (*), (**), (***): *p* < 0.01, 0.002, 0.0002).

**Figure 4:**
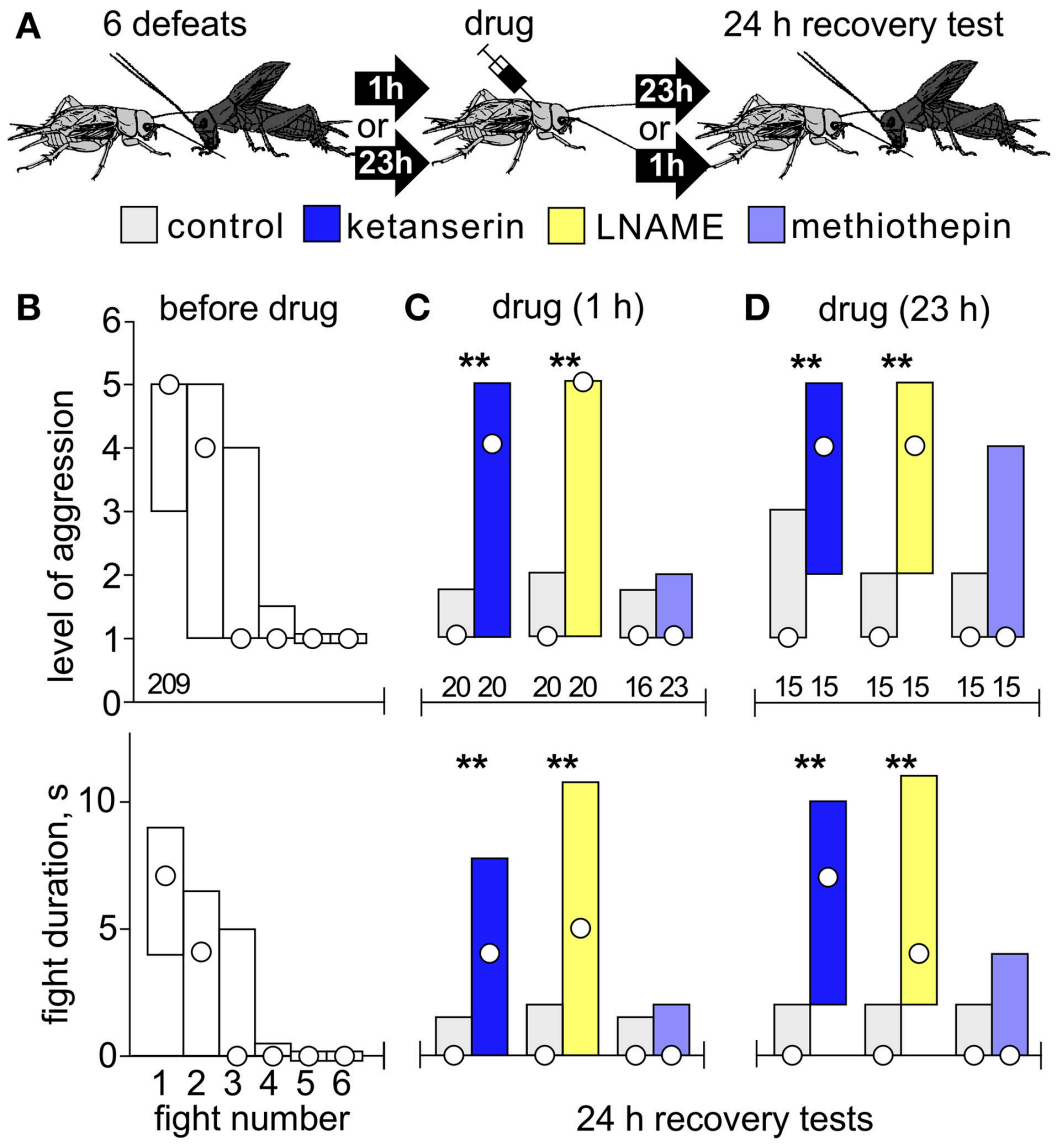
Effects of drugs after chronic social defeat. **(A)** Procedure: Crickets were first matched 6 times at 1 h intervals against hyper-aggressive opponents, then treated either 1 h or 23 h later with drug, and their aggressiveness evaluated 24 h after the last defeat (recovery test). **(B)** Bar charts giving the level of aggression (top) and fight duration (right, circle: median, bars: interquartile range, IQR) before drug treatment (white bars). **(C)** As in B but 24 h later for crickets that received LNAME (dark blue bars), ketanserin (yellow bars), methiothepin (pale blue bars) 1 h after multiple defeat. **(D)** As for **C**, but 23 h after multiple defeat. Controls received vehicle or DNAME to control for LNAME (gray bars).

1. Separate cohorts of test crickets were pretreated with drug and then matched at a first fight against a hyper-aggressive opponent 60 min later (exception: AMTP 24 h later) and then once more against the previous opponent either 15, 30, 60, or 180 min later to evaluate loser recovery (Figure 1A).
2. Separate cohorts of crickets were matched at a first fight against a hyper-aggressive opponents and then again either 6, or 2 times in succession at 1 h intervals (multiple defeat), and then finally once more after 24 h (recovery test; Figures 2A, 3A). The hyper-aggressive opponents were swapped at each match to preclude defeated crickets adapting their behavior toward familiar opponents (Trannoy and Kravitz, 2017).
3. Here, untreated crickets were first matched 6 times in succession at 1 h intervals (multiple defeat) and subsequently treated with drug, either 1 or 23 h after the last defeat, and then tested once more 24 h after the last defeat (recovery test; Figure 4A).

### Data analysis

Our analysis is based on 1991 test crickets. Each was used for only one experiment. Statistical tests were performed using Prism 6 (GraphPad, La Jolla, CA, USA). The median and the interquartile range (IQR) were calculated for non-parametric data sets and non-parametric tests were also performed on fight duration since the data failed D'Agostino and Pearson omnibus normality tests, even after log transformations. The Mann-Whitney *U*-test was used to test for significant differences in the distributions between two unpaired data sets. An alpha value of *P* < 0.05 was considered significant (^*^, ^**^, ^***^ *p* < 0.05, 0.01, 0.001 respectively). In the multiple defeat experiments we tested the hypothesis whether or not 6, or 2 previous defeats lead to long-term aggressive depression, and how this is influenced by drugs. For completeness, we also show the data for the 6 previous fights, and since these are repeated comparisons of the same animal groups we applied the Bonferroni correction to alpha for 5 multiple comparisons (^*^p < 0.01). In one experiment (Figure S2), 3 treatments were compared to control, and we applied the Kruskal-Wallis test with Dunn's multiple comparisons.

## Results

### 5HT, initial fights, and loser depression

In our first experiment, we pre-treated crickets with drug and evaluated their first fights, and then a second fight at different times after defeat against hyper-aggressive opponents (Figure 1A). At the first fight, controls typically escalated to physical interactions that lasted several seconds (e.g., AMTP-control, level: median 5, IQR 3-5; duration: median 6 s, IQR 3-9.75, *n* = 80). Compared to controls, the level and duration of aggression at the first fight was not significantly different for crickets pretreated with AMTP (Figure 1B, *U*-test: *p*-level = 0.72; *p*-duration = 0.76), the receptor blockers ketanserin (Figure 1C, *U*-test: *p*-level = 0.33; *p*-duration = 0.94) or methiothepin (Figure 1D, *U*-test: *p*-level = 0.23; *p*-duration = 0.18), the precursor 5HTP (Figure 1E, *U-*test: *p*-level = 0.66; *p*-duration = 0.64) or the re-uptake inhibitor fluoxetine (Figure 1F, *U*-test: *p*-level = 0.26; *p*-duration = 0.40). Subsequently, 15 min after defeat, all groups of vehicle treated losers were non-aggressive and tended to retreat from the hyper-aggressive opponent (e.g., AMTP-controls: median level 1, IQR 1-2, *n* = 20; see also Stevenson et al., 2005). The aggressiveness of drug treated crickets 15 min after defeat was not significantly different to their respective controls, except for those that received ketanserin, which were significantly more aggressive at the 15 min trial (*U*-test: *p*-level = 0.036; *p*-duration = 0.019). This fitted the trend that ketanserin and AMTP induced earlier recovery from social defeat, whereas recovery was suppressed by 5HTP and fluoxetine. For example, 30 min after defeat, ketanserin treated crickets were significantly more aggressive than controls (*U*-test: *p*-level = 0.003; *p*-duration = 0.0011). On the other hand, fluoxetine treated crickets still showed significantly depressed aggression (median level 1, IQR 1-2, *n* = 31) compared to controls 180 min after defeat (median level 5, IQR 2-5, *n* = 31, *U*-test: *p*-level < 0.001; *p*-duration < 0.001). A similar, but less pronounced trend was also evident for 5HTP-treated crickets (Figure 1E). Contrasting this, methiothepin treated crickets at the 180 min trial tended to be less aggressive than controls (*U-*test: *p*-level = 0.025; *p*-duration = 0.027), indicating that this 5HT receptor blocker may act to dampen loser recovery.

### 5HT and chronic social defeat

We next analyzed the influence of pre-treatment with serotonergic drugs on the acquisition of longer-term depression of aggression after chronic social defeat (Figure 2). Whereas controls typically regain their aggressiveness within 3 h of a single defeat (Figure 1), or 24 h after two defeats, after 6 defeats they typically retreated at the 24 h trial (e.g., AMTP-controls: median level 1, IQR 1-1, *n* = 20, Figure 2B). Drug treatment again indicated that 5HT suppresses aggression specifically after social defeat. For example, while controls became progressively less aggressive with each encounter, ketanserin treated crickets remained aggressive, and showed significantly higher aggression and fight duration at the 4th, 5th, and 6th encounter (Figure 2C, e.g., 6th fight: median level 5, IQR 1.75-5, median duration 6 s, IQR 0.5-9.75, *n* = 20, *U*-test: *p*-level < 0.001; *p*-duration < 0.001). Furthermore, ketanserin significantly blocked the acquisition of longer-term aggressive depression so that 24 h after 6 defeats the test crickets were significantly more aggressive than controls (ketanserin: median level 5, IQR 1.25-5, *n* = 20; control: median level 1, IQR 1-2, *n* = 19, *U*-test: *p*-level = 0.0026; *p*-duration < 0.001). Compared to this, there was no difference between ketanserin and control 24 h after 2 defeats (*U*-test: *p*-level = 0.435; *p*-duration = 0.625). AMTP had essentially the same effect as ketanserin, though less pronounced (significant differences indicated in Figure 2B). The 5HT receptor blocker methiothepin, however had no significant effect on the level of aggression or duration at any trial compared to control (Figure 2D). For example, 24 h after 6 defeats methiothepin treated crickets exhibited depressed aggression, and showed no significant difference to control (*U*-test: *p*-level = 0.907; *p*-duration = 0.791).

The precursor of 5HT and its re-uptake inhibitor, in contrast, increased susceptibility to social defeat stress. First, and as also indicated in Figures 1E,F, crickets that received 5HTP or fluoxetine were significantly less aggressive than controls at the second fight, 1 h after the first defeat (e.g., fluoxetine compared to vehicle, *U*-test: *p*-level < 0.001; *p*-duration < 0.001). Secondly, whereas 24 h after 2 defeats the control recovered from defeat (e.g., fluoxetine control: median level 4, IQR 1-5), those that received 5HTP or fluoxetine showed significantly depressed aggression (e.g., fluoxetine, *U*-test: *p*-level < 0.001; *p*-duration < 0.001).

### Nitric oxide and other amines

In contrast to the 5HT inhibitors AMTP, ketanserin and methiothepin, and confirming our earlier study (Stevenson and Rillich, 2015) pre-treatment with the NO-synthesis inhibitor LNAME led to a significant increase in aggression at the first fight (e.g., *U*-test compared to DNAME: *p*-duration = 0.0035; Figure 3B). Otherwise, LNAME's effect matched that of ketanserin and AMTP, but was even more pronounced. For example, whereas the alleviating effect of ketanserin on loser depression first became evident after 4 successive defeats (Figure 2C), LNAME treated crickets showed recovery 1 h after the first defeat (median level 5, IQR 3.25-6, *U*-test compared to DNAME: *p*-level < 0.001; *p*-duration < 0.001**)**, and no signs of loser depression with subsequent defeats (Figure 3B). Furthermore, compared to control, and as found for ketanserin, LNAME treated crickets showed no sign of long term aggressive depression 24 h after 6 defeats (median level 5, IQR 4-5.75, n = 16, *U*-test compared to DNAME: *p*-level < 0.001; *p*-duration < 0.001).

Interestingly, the insect dopamine (DA) receptor antagonist fluphenazine had a similar effect as 5HT agonists, in that it increased susceptible to social defeat stress (Figure 3C). As for all serotonergic drugs tested, fluphenazine had no significant effect on aggression at the first fight (*U*-test: *p*-level = 0.985; *p*-duration = 0.987). However, as for the agonists 5HTP and fluoxetine (Figures 2E,F), fluphenazine treated crickets were significantly less aggressive than controls at the second fight, 1 h after their first defeat (*p*-level < 0.001; *p*-duration < 0.001). Finally, and again as seen after receiving 5HTP or fluoxetine (Figures 2E,F), fluphenazine treatment resulted in significantly depressed aggression 24 h after only 2 defeats (*U*-test: *p*-level < 0.001; *p*-duration < 0.001). We found no significant effects of the insect octopamine (OA) receptor antagonist epinastine on aggression expressed at any of the contests staged for the multiple defeat paradigm (Figure 3D, e.g., second fight: *U*-test, *p*-level = 0.360; *p*-duration = 0.448; 24 h after 2 defeats: *U*-test, *p*-level = 0.281; *p*-duration = 0.180).

### Effect of drugs applied after multiple defeat

To check whether ketanserin and LNAME block long term aggressive depression after multiple defeat (Figures 2, 3) by simply facilitating earlier recovery from each defeat, we tested their effects when given 1 and 23 h after multiple defeat (Figure 4). Before drug, crickets again showed progressively declining aggressiveness with each defeat, so that at the 6th fight they typically retreated (median level 1, IQR 1-1, median duration 0s, IQR 0-0). When tested 24 h later, controls (DMSO and DNAME) still showed reduced aggression, as did those that received methiothepin (Figures 4C,D). Contrasting this, both ketanserin and LNAME prohibited acquisition of long term depression, regardless of whether the drugs were given 1 h (Figure 4C) or 23 h (Figure 4D) after multiple defeat (*U*-tests compared to respective control, ketanserin at 1 h: *p*-level = 0.0031; *p*-duration = 0.0016; ketanserin at 23 h: *p*-level = 0.0037; *p*-duration = 0.0034; LNAME at 1 h: *p*-level = 0.0015; *p*-duration = 0.0013; LNAME at 23 h: *p*-level = 0.0048; *p*-duration = 0.0034).

## Discussion

This study provides novel insight into the natural behavioral role of 5HT in insect aggression, with potential parallels to vertebrates. We propose that 5HT is released specifically after social defeat to maintain depressed aggressive behavior in losers for a progressively longer period with successive defeats, resulting in long term behavioral depression, analogous to the chronic-defeat stress syndrome in mammals (Hammels et al., 2015; De Boer et al., 2016; Trainor et al., 2017). To evaluate the full aggressive potential of each individual test cricket, we recorded how they escalate (level of aggression) and persist (fight duration) against standard hyper-aggressive opponents that always won. This is in essence similar to the intruder-resident paradigm in rodents, where small intruders are matched against more aggressive residents (Trainor et al., 2017). Drugs were applied at relatively high concentrations (Table 1) in order to overcome the brain's sheath (see Stevenson et al., 2005). Despite this, the effective dosage in nervous tissue can be expected to be in the physiological range, since each drug had selective effects on aggression, without adversely affecting general motility, and we were able to discriminate the specific actions of different and even closely related transmitters (e.g., Figures 2, 3; see also Rillich and Stevenson, 2014) and in some instances, even receptor subtypes (below).

None of the tested serotonergics administered before the tournament influenced aggression at the first fight (Table 1; Figures 1, 2). This conflicts with reports that 5HT typically promotes aggression in invertebrates (Kravitz, 2000; Dierick and Greenspan, 2007; Johnson et al., 2009; Alekseyenko et al., 2010; Bubak et al., 2014). We suspect that this discrepancy may at least partly reflect differences in drug concentration and application (acute vs. chronic), that can differentially affect different 5HT receptor subtypes, as shown for 5HTP (Pranzatelli, 1988). In our hands, a single dose of 5HTP (20 μl/5 mM) failed to influence a cricket's initial fighting behavior (Figure S1), whereas the 100-fold dose (100 μl/100mM) increased some elements of aggression (fight duration), but reduced others (threat behavior, attack frequency), without affecting win chances (Dyakonova and Krushinsky, 2013). On the other hand, chronic treatment by feeding for 4 days on 5HTP led only to increased aggression in fruit flies (20 mM, see Dierick and Greenspan, 2007) and stalk-eyed flies (3% ≈ 135 mM, see Bubak et al., 2014). However, feeding *Drosophila* for 3-4 days with comparatively low drug concentrations (3 mM) confirmed that 5HT can promote aggression in socially naive *Drosophila*, and it was revealed that 5HT elevates aggression specifically via a 5HT_1A_ like receptor (Johnson et al., 2009). Furthermore, acute genetic activation of a subset of 5HT neurons in *Drosophila* heightens aggression, and one pair seems necessary for aggressive escalation, which seem to act *via* 5HT_1A_ receptors to inhibit aggression-suppressing follower neurons (Alekseyenko et al., 2010, 2014). In view of this, 5HT might under circumstances that remain to be revealed also promote aggression in socially naive crickets. However, as outlined below, our data suggests that its main action is to dampen the normal recovery of aggression after social defeat.

Acute treatment with fluoxetine prohibited the normal recovery of aggression after a single defeat (Figure 1F), and increased susceptibility to chronic social defeat, in that only 2 defeats sufficed to induce longer term aggressive depression (Figure 2F). The effects of acute fluoxetine treatment are generally thought to result from blocking the transporter for removing 5HT after release in mammals (Morrison and Melloni, 2014) and also insects, though less effectively (Corey et al., 1994). Fluoxetine can also, however, increase catecholamine levels, particularly after chronic administration (Bymaster et al., 2002), and then have antidepressant effects, including reduced defeat-induced pathophysiology in rodents (Bauer, 2015; Hammels et al., 2015). Even so, since 5HTP had the same effect as fluoxetine on cricket aggression (Figures 1E, 2E), and the latter's action was blocked by the 5HT receptor antagonist ketanserin (Figure S2), fluoxetine probably also increases endogenous 5HT in crickets. Supporting this, aggressive depression in losers was reduced after AMTP (Figure 1B), and even more effectively by the 5HT receptor blocker ketanserin (Figure 1C). These inhibitors also prevented longer term aggressive depression after chronic defeat (Figures 2B,C). This is not simply due to the drugs, which were applied before the tournament, preventing loser depression from occurring in the first place, since ketanserin also prohibited long term depression even when given 23 h after multiple defeat (Figure 4D). This indicates that 5HT levels are elevated for at least a day after experiencing multiple defeat.

Contrasting ketanserin, methiothepin tended to prolong loser depression (Figure 1D), which we think is due to effects on a different 5HT receptor subtype. Insects and vertebrate 5HT receptors are phylogenetically and functionally related, but have different pharmacological profiles (Vleugels et al., 2015). Crickets express two 5HT_1_, two 5HT_2_, one 5HT_7_ receptor (Watanabe et al., 2011; Watanabe and Aonuma, 2012), but they are not yet fully pharmacologically characterized. Ketanserin is regarded as selective for insect 5HT_2_ receptors (Johnson et al., 2009), whereas methiothepin is less selective (Vleugels et al., 2015), but is considered to block all subtypes in combination with ketanserin. In honeybees, for example, methiothepin blocks all 5HT receptors except 5HT2B, which is selectively blocked by ketanserin (Thamm et al., 2013; Tedjakumala et al., 2014). We therefore propose, that 5HT decreases aggression after defeat in crickets *via* a 5HT2 type receptor. Notably, and in agreement with our observations on crickets, ketanserin had no effect on aggression in socially naive fruit flies (Johnson et al., 2009).

In mammals, overt aggression is depressed mainly *via* 5HT_1A_ receptors in the dorsal Raphe nucleus, and promoted by 5HT_2_ receptors (De Boer and Koolhaas, 2005; Morrison and Melloni, 2014; Olivier, 2015; Carhart-Harris and Nutt, 2017). However, in the amygdala 5HT_1A_ agonist decreases acquisition of conditioned defeat (Morrison and Cooper, 2012), whereas 5HT_2A_ agonists promote post defeat anxiety and 5HT_2A_ antagonists reduced it (Bauer, 2015; Clinard et al., 2015). Thus, 5HT_2_ like receptors seem to maintain depressed aggressive behavior after social defeat in both crickets and mammals.

Since the aggression depressing effect of 5HT was only evident in losers, it seems to depend on the animal's subordinate social status. In crayfish, changes in social status entail changes in neuronal circuits and possibly 5-HT receptors (Issa et al., 2012), but this need not be the case in crickets. An alternative, or at least complementary possibility, is that the action of 5HT in losers depends on prior activation of the neurotransmitter pathway that controls the initial decision to retreat. In crickets, the decision to flee and subsequent loser depression is initiated by nitric oxide (NO) and does not require 5HT (Stevenson and Rillich, 2015; Rillich and Stevenson, 2017). Here we showed that blocking NO synthesis with LNAME either before (Figure 3B) or after (Figure 4) multiple defeats prohibited long term aggressive depression even more effectively than ketanserin. Notably, and in contrast to blocking 5HT, blocking NO increases aggression at the first fight (Stevenson and Rillich, 2015, and Figure 3B). Since 5HT's dampening effect on cricket aggression requires prior social defeat, it depends on NO, but it needs to be tested whether or not NO acts directly on serotonergic neurons. In mammals, disruption of NO production also leads to substantially increased aggression, possibly be interacting with 5HT, but the relationship is unclear (Bedrosian and Nelson, 2014). To our knowledge it is not known if NO influences defeat induced depression in mammals.

We have previously shown that both OA and DA restore aggression in losers after a single defeat, whereby DA, but not OA, is actually necessary for natural recovery (Rillich and Stevenson, 2014). Here we investigated how the DA receptor antagonist fluphenazine influenced aggression after multiple defeats and found that it had the same effect as elevating 5HT. Thus, as for 5HTP and fluoxetine, fluphenazine had no effect on aggression at the initial fight, but increased the susceptibility to chronic defeat in that only 2 successive defeats established long term aggressive depression (Figure 3C). In contrast, blocking receptors for OA, which is recognized as an insect stress hormone (Adamo and Baker, 2011) and has promoting effects on cricket aggression (reviews: Stevenson and Rillich, 2016, 2017), had no effect on post-defeat aggression (Figure 3D). We suspect, therefore, that 5HT's dampening effect on loser aggression may result from inhibition of DA, but this needs to be specifically tested. In mammals, 5HT suppresses aggression mainly by inhibiting neurons that release and or respond to arginine-vasopressin (Morrison and Melloni, 2014). However, DA may also be involved. Social defeat increases activity of DA neurons in the mesolimbic system (Laman-Maharg and Trainor, 2017), where DA plays a central role in reward (O'Connell and Hofmann, 2011) and in mediating anhedonia due to social defeat (Hammels et al., 2015).

In summary, our data call for a re-evaluation of the role of 5HT in invertebrate aggression. In contrast to most invertebrate studies, we found no evidence that 5HT acts to increase aggression in socially naïve crickets. However, in view of our finding that methiothepin tended to enhance aggression in losers (Figure 1D), we still think that 5HT may under behavioral circumstances that remain to be discovered, have a natural aggression promoting effect, as suggested by work of Dyakonova and Krushinsky (2013), for example via 5HT_1_-like receptors as in *Drosophila* (Johnson et al., 2009; Alekseyenko et al., 2014), but this needs to be tested in crickets with more selective drugs. Nonetheless, our experiments, particularly those with fluoxetine and ketanserin, indicate that 5HT is released after social defeat and acts *via* 5HT_2_-type receptor to maintain the state of depressed aggressiveness characteristic for subordinates. This contrast the earlier suggestion that “a decrease in serotonergic activity may be functionally important for the control of loser behavior” (Dyakonova and Krushinsky, 2013),” but complies with the finding that the brain content of 5HT is reduced after defeat (Murakami and Itoh, 2001), when this is considered as a consequence, rather than cause of defeat. Taken together, the control of post-defeat aggression is surprisingly similar to that in mammals (Bauer, 2015; Clinard et al., 2015) and possibly also crustaceans (Bacque-Cazenave et al., 2017). Contrary to current understanding in mammals (Bedrosian and Nelson, 2014), however, it seems that in crickets NO release is a pre-requisite for 5HT's inhibitory action on the recovery from social defeat stress, which in turn may result from inhibition of DA. This, however, remains to be experimentally verified.

## Ethics statement

The animals used in this study (invertebrates, insects, crickets: *Gryllus bimaculatus*), are exempt from any need to obtain any special permissions, all animals were from a breeding stock, and none removed from their natural environment. All treatments complied with the Principles of Laboratory Animal Care and the German Law on the Protection of Animals.

## Data availability statement

The raw data supporting the conclusions of this manuscript will be made available by the authors, without undue reservation, to any qualified researcher.

## Author contributions

JR and PS conceived and designed the experiments. JR Performed the experiments. JR and PS analyzed the data. PS contributed reagents materials analysis tools. PS and JR wrote the paper.

### Conflict of interest statement

The authors declare that the research was conducted in the absence of any commercial or financial relationships that could be construed as a potential conflict of interest.

## Supplementary material

Figure S1 Dose dependent effects of fluoxetine **(A)** and 5HTP **(B)**. Plots of level of aggression exhibited by crickets at their first fight against hyper-aggressive opponents and 3 h after defeat (symbols: median, bars interquartile range, *n* > 16 for each).

Figure S2 Ketanserin blocks effect of fluoxetine. **(A)** Procedure: Test cricket received vehicle or drug 1 h before their first fight against hyper-aggressive opponents, which they lost, and once more 1 h after defeat. **(B)** Level of aggression, **(C)** Fight duration. Significant differences are given as *p*-values from Kruskal-Wallis tests, and differences between groups from Dunn's multiple comparisons are indicated by asterisks: ^**^ *p* < 0.01, n.s. not significant. Note that fluoxetine no longer prohibits post-defeat recovery (red bars) when given together with the 5HT receptor blocker ketanserin (red-hatched bars).
