# Supplementary figures and images for: Serotonin Mediates Depression of Aggression After Acute and Chronic Social Defeat Stress in a Model Insect
Source: Front Behav Neurosci. 2018 Oct 8;12:233. doi: 10.3389/fnbeh.2018.00233 (PMC6186776; doi:10.3389/fnbeh.2018.00233)

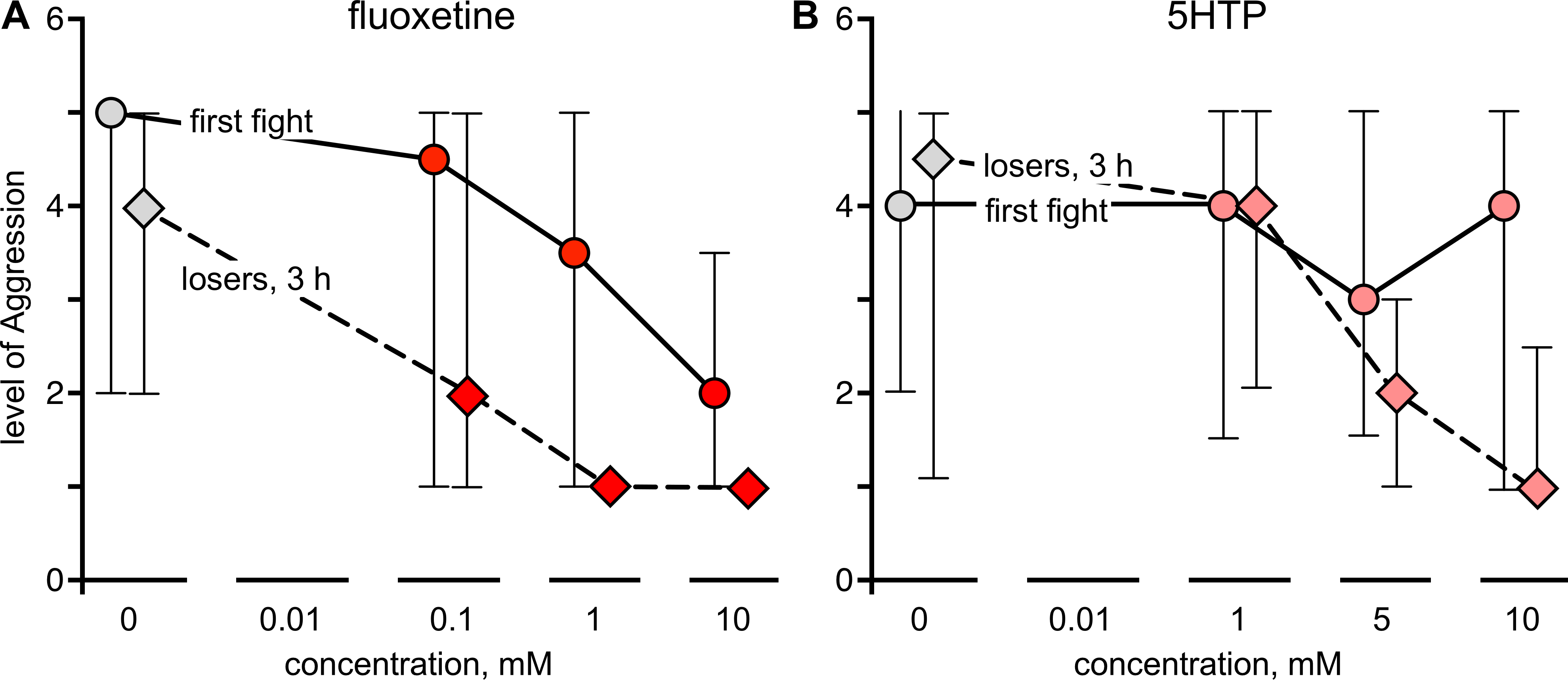

Supplement: Figure S1 — Dose dependent effects of fluoxetine (A) and 5HTP (B). Plots of level of aggression exhibited by crickets at their first fight against hyper-aggressive opponents and 3 h after defeat (symbols: median, bars interquartile range, n > 16 for each). [file Image_1.TIF]

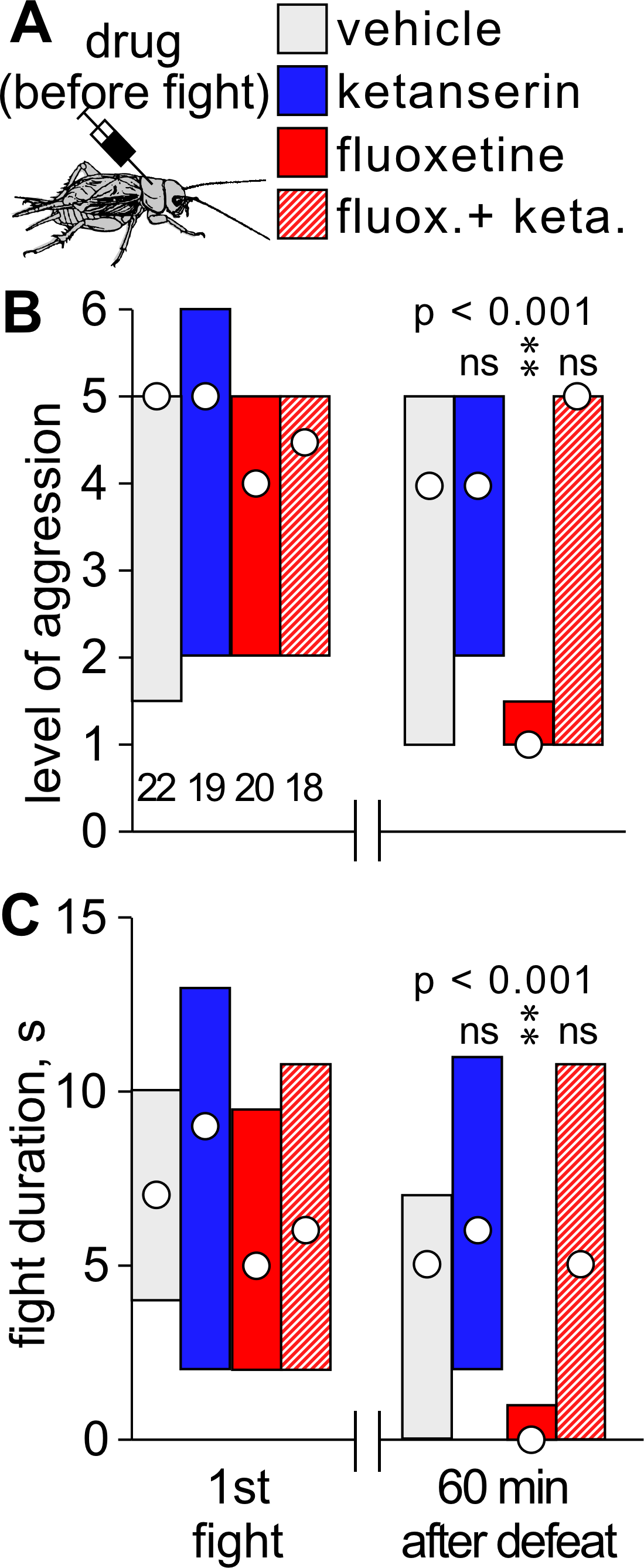

Supplement: Figure S2 — Ketanserin blocks effect of fluoxetine. (A) Procedure: Test cricket received vehicle or drug 1 h before their first fight against hyper-aggressive opponents, which they lost, and once more 1 h after defeat. (B) Level of aggression, (C) Fight duration. Significant differences are given as p-values from Kruskal-Wallis tests, and differences between groups from Dunn's multiple comparisons are indicated by asterisks: ** p < 0.01, n.s. not significant. Note that fluoxetine no longer prohibits post-defeat recovery (red bars) when given together with the 5HT receptor blocker ketanserin (red-hatched bars). [file Image_2.TIF]
